# Associations of maternal pre-pregnancy obesity and excess pregnancy weight gains with adverse pregnancy outcomes and length of hospital stay

**DOI:** 10.1186/1471-2393-11-62

**Published:** 2011-09-06

**Authors:** Abdullah A Mamun, Leonie K Callaway, Michael J O'Callaghan, Gail M Williams, Jake M Najman, Rosa Alati, Alexandra Clavarino, Debbie A Lawlor

**Affiliations:** 1 School of Population Health, The University of Queensland, Brisbane, Australia; 2 Royal Brisbane and Women's Hospital, Brisbane, Australia; 3 School of Medicine, The University of Queensland, Brisbane, Australia; 4 Mater Children's Hospital, and The University of Queensland, Brisbane Australia; 5 School of Pharmacy, The University of Queensland, Brisbane, Australia; 6 MRC Centre for Causal Analyses in Translational Epidemiology, Department of Social Medicine, University of Bristol, UK

**Keywords:** pregnancy outcomes, gestational weight gain, pre-pregnancy obesity, health care

## Abstract

**Background:**

It is relatively less known whether pre-pregnancy obesity and excess gestational weight gain (GWG) are associated with caesarean delivery, pregnancy complications, preterm birth, birth and placenta weights and increased length of postnatal hospital stay.

**Methods:**

We used a population-based cohort of 6632 women who gave birth in Brisbane, Australia, between 1981 and 1983. The independent associations of pre-pregnancy obesity, GWG and institute of medicine (IOM) categories of combined pre-pregnancy BMI and GWG with outcomes were examined using multivariable regression (for continuous outcomes) and multivariable multinomial regression (for categorical outcomes) models.

**Results:**

We found women who were obese prior to pregnancy and women who gained excess weight during pregnancy were at greater risk for a pregnancy complications (OR: 2.10; 1.74, 2.54; age adjusted model), caesarean section (OR 1.29; 1.09, 1.54), higher birth weight difference (206.45 gm; 178.82, 234.08) and greater placental weight difference (41.16 gm; 33.83, 48.49) and longer length of hospital stay. We also found that mothers who gained inadequate weight or were underweight before pregnancy were at greater risk of preterm birth (2.27; 1.71, 3.00), lower risk of pregnancy complications (0.58; 0.44, 0.77) and had lower birth (-190.63;-221.05,-160.20) and placental (-37.16; -45.23,-29.09) weights. Results indicate that all associations remain consistent after adjustment for a range of potential confounding factors with the exception of the association between pre-pregnancy obesity and hospital stay.

**Conclusions:**

Pre-pregnancy obesity or excessive GWG are associated with greater risk of pregnancy complications, caesarean delivery and greater birth and placenta weight. Excess GWG is associated with a longer stay in hospital after delivery, independent of pre-pregnancy BMI, pregnancy complications and caesarean delivery. In addition to pre-pregnancy obesity, it is vital that clinical practice considers excess GWG as another indicator of adverse pregnancy outcomes.

## Background

Consistent with broader population trends, the prevalence of overweight and obesity is increasing rapidly among obstetric populations all over the world [1,2]. One in three Australian women aged 25-35 years are overweight or obese (i.e. BMI > 24 kg/m^2^) [3], 44% of USA women in the 18-49 age group are overweight or obese [4] and a study from the United Arab Emirates reported that about 40% of married women are obese [5]. A study from the North of England found that obesity levels (BMI > = 30 kg/m^2^) in women at a booking clinic increased from 9.9% in 1990 to 16.0% in 2004 [6]. Women who are overweight or obese at the start of pregnancy are at increased risk of hypertensive disorders of pregnancy [7,8], gestational diabetes [9], delivery complications such as prolonged delivery and higher rates of caesarean sections [10]. In a recent review and meta analysis it has also been suggested that overweight and obese women have increased risks of preterm births, after accounting for publication biased [11]. Furthermore, studies consistently reported that pre-pregnancy BMI positively associated with infant birth weight [12,13]. Few studies have also reported that complications due to obesity lead to excess health care service use, including increased length of hospital stay, during and immediately after pregnancy [14-17].

In addition to established risks associated with pre-pregnancy overweight or obesity, there has been increased interest in the potential adverse consequences of excess weight gain in pregnancy, irrespective of the woman's size at the start of pregnancy [18,19]. Given that more than one-third of mothers gain excess weight during pregnancy [20-22], two key issues from a health policy perspective, are (i) to determine whether there is a link between excessive weight gain and adverse pregnancy outcomes including hypertensive disorder of pregnancy, gestational diabetes, caesarean delivery, premature birth, birth weight and placenta weight and (ii) whether this excessive weight gain increased health care utilization. A recent study based on the Danish National Birth cohort (a very large sample of nearly 61,000 mothers and their infants) found that independent of pre-pregnancy BMI, excess weight gain in pregnancy was associated with increased risk of large for gestational age infants, caesarean section delivery, low apgar score and postnatal weight retention in the mother [23]. These adverse perinatal outcomes might be expected to result in excess health care utilization associated with gestational weight gain. However, to our knowledge no previous study has examined whether excess weight gain in pregnancy is associated with increased length of postnatal hospital stay. Given the independent association of weight gain in pregnancy with adverse perinatal outcomes reported in the Danish National Birth cohort our hypothesis is that independent of their BMI at the start of pregnancy, women who gain more weight in pregnancy will experience greater adverse pregnancy outcomes and will have longer postnatal hospital stays than women who gain less weight in pregnancy. Furthermore, we hypothesise that the association between excessive weight gain and postnatal hospital stay will be mediated (and hence attenuate towards the null) by complications of pregnancy and caesarean section delivery.

## Methods

### The study

The data we used were from the Mater-University Study of Pregnancy and its outcomes (MUSP). MUSP is a prospective birth cohort study of 7,223 women, and their offspring, who received antenatal care at a major public hospital in Brisbane, Australia, between 1981 and 1983 and delivered a live singleton child who was not adopted before leaving hospital [24,25]. Multiple births were excluded from the analyses presented here as by definition, they will gain more weight than singleton births and are likely to have a longer hospital stay irrespective of pregnancy complications. These mothers and their offspring have been followed-up prospectively, with assessments being conducted when their offspring were 6 months, 5, 14 and 21 years. In this study the main analyses are restricted to 6528 mothers for whom data was available on both exposures and outcomes. Written informed consent from the mothers was obtained at all data collection phases of the study. Ethics committees at the Mater Hospital and the University of Queensland approved each phase of the study. Full details of the study participants and measurements have been previously reported [24,25].

### Measurements

#### Pre-pregnancy BMI

Maternal pre-pregnancy BMI was calculated as weight in kg divided by height in meters squared using self-reported pre-pregnancy weight, recorded at baseline from maternal questionnaires, and height measured at the first antenatal clinic visit. At the first antenatal clinic visit women were asked to report their pre-pregnancy weight; and were also weighed at this clinic. There was a high correlation between these two measures (Pearson's correlation coefficient = 0.95). BMI was categorized into normal (< 25 kg/m^2^), overweight (25-29 kg/m^2^) and obese (> = 30 kg/m^2^) using the WHO classification of BMI cut-offs [26].

#### Gestational Weight Gain (GWG)

A recent study that examined different methods of calculating GWG concluded that none of the methods were distinctively superior with respect to neonatal outcomes (e.g. birth size and gestational age). A simple difference (end of pregnancy weight minus beginning of pregnancy weight) was the superior measure for maternal weight retention at 6 months and for maternal weight retention at later time points (up to 36 months), the area under the curve method was superior [27]. In this study, we examined associations with three measures of GWG - a simple difference (maximum weight in pregnancy minus pre-pregnancy weight), average weekly gain (the simple difference divided by gestational age) and Institute of Medicine (IOM) [28] categories. Using a simple difference of pregnancy weight gain, the results were identical to those using average weight gains per gestational week. Therefore we have opted to present only results for average weight gain per gestational week and for IOM categories.

Weight gain during pregnancy was calculated from maximum weight measured in pregnancy and the mother's self-reported pre-pregnancy weight. Maximum weight in pregnancy was abstracted from the medical chart by an obstetrician associated with the MUSP. We found 53 women who did not appear to change weight during pregnancy and a further 75 who appeared to gain more than 30 kg. While it is known why some women do not gain weight in pregnancy and others gain excessive amounts, such extreme changes are likely to be related to uncommon pathologies, which we did not want to have a major influence on our findings, and therefore we excluded these 128 women from all analyses.

We calculated *total gestational weight *gain as the difference between maximum recorded weight gain during pregnancy and self-reported pre-pregnancy weight (determined at the first antenatal visit). We calculated *average weight *gain during pregnancy as this maximum weight minus her pre-pregnancy weight divided by gestational age. In our analyses of weight gain per gestational week, we scaled this to provide differences in length of hospital stay for a 0.10 kg/weeks of gestation weight gain. This was chosen as a plausible weight change in pregnancy and is consistent with a previous publication from this cohort [22]. We also categorised women as having gained inadequate, adequate, or excess weight according to IOM guidelines [28] (see additional file 1 table S1 for the derivation of IOM categories). This guideline recommended that obese women should not gain more than 11.5 kg but no upper limit was provided. For this study, we assume that women who gained more that 11.5 kg during the pregnancy are in excess weight gain category. Recently IOM reviewed their guidelines [28] and recommended rates of weight gain in 2^nd ^and 3^rd ^trimester as well. As we do not have the record of trimester specific weight gain, we could not extend our analysis for this finer category [28].

#### Pregnancy outcomes

For this study, we considered pregnancy complications or high risk pregnancy (hypertensive disorder of pregnancy or gestational diabetes), method of delivery, gestation or preterm birth (normal, premature), birth weight (measured in grams) and placental weight (measured in grams) as pregnancy outcomes. Hypertensive disorders in pregnancy (HDP) were diagnosed at birth by a consultant obstetrician and defined as a diastolic BP over 90 mmHg on at least two occasions beyond 20 weeks gestation associated with proteinuria and/or excessive fluid retention (defined as generalized oedema including the face and hands and excessive weight gain) [29]. For the purpose of this study, all delivery methods were grouped into three categories: normal delivery, caesarean delivery and others (forceps, ventouse, assisted breech and combined methods). Preterm birth was defined as normal if gestation was more than 36 weeks and premature if gestation was 21 to 36 weeks. Birth weight, placental weight and methods of delivery were obtained from the obstetric records.

#### Length of hospital stay

Length of time spent in hospital (by number of days) immediately after delivery was calculated by subtracting the date of delivery from the date of discharge from the hospital. Both date of delivery and date of discharge were obtained from the obstetric medical records.

#### Confounders

The potential confounders are selected on the basis of a priori knowledge [30] of their association with exposure and outcome. Available potential confounders were maternal age at birth (in years), maternal educational attainment (did not complete secondary school, completed secondary school, completed further/higher education), parental ethnic origins (White, Asian or Aboriginal/Islander), parity (1, 2, 3 or more) maternal pre-pregnancy consumption of cigarettes (none, 1-19 or 20 or more per day), alcohol (abstainer, light drinker or 1+ glass per day) and maternal depression (depressed vs. non-depressed) using the scale Delusions Symptoms-States Inventory: State of Anxiety and Depression (DSSI/SAD) [31] during pregnancy was used, all of which may affect both exposures and outcomes.

### Statistical analyses

We used analysis of variance and F tests to compare mean values and a chi squared test for categorical values of maternal characteristics by IOM categories of weight gain in pregnancy (additional file 1 tables S2 and S3) and maternal pre-pregnancy BMI categories (additional file 1 table S3). The odds of being an adverse pregnancy outcome was estimated using multinomial (when outcome was three categories- e.g. method of delivery) or logistic (when outcome was dichotomous- e.g. gestation and high risk pregnancy) regressions (table 1). The mean difference of birth weight (in grams) and placenta weight (in grams) were estimated using multiple regression (table 2). The distribution of length of hospital stay followed an approximately normal distribution (results available from author on request). The mean difference of length of stay in hospital from delivery to discharge by maternal gestational weight gain, pre-pregnancy BMI categories and IOM recommendations was estimated using multiple linear regression (Tables 3 and 4 and additional file 1 figure S1). A series of models were performed to test differential effects of confounders and mediators (see footnotes of tables and figure for details).

**Table 1:** Odds (95% confidence interval) of being caesarean and other methods of pregnancy delivery, preterm births and high risk pregnancy by IOM, pre-pregnancy BMI categories and gestational weight gain per 0

	Method of delivery^a^				Preterm birth^b^		Pregnancy complications^c^	
	Caesarean		Other					
	**Age adjusted**	**Fully Adjusted^§^**	**Age adjusted**	**Fully adjusted**^**§**^	**Age adjusted**	**Fully adjusted**^**¥**^	**Age adjusted**	**Fully adjusted**^**¢**^

**IOM**								
Inadequate	0.89 (0.73,1.09)	0.79 (0.64,0.97)	0.79 (0.64,0.98)	0.78 (0.63,0.98)	2.27 (1.71,3.00)	2.41 (1.81,3.20)	0.58 (0.44,0.77)	0.58 (0.44,0.77)
Adequate	1.00	1.00	1.00	1.00	1.00	1.00	1.00	1.00
Excess	1.29 (1.09,1.54)	1.34 (1.12,1.60)	1.25 (1.04,1.49)	1.24 (1.03,1.50)	0.63 (0.44,0.89)	0.53 (0.37,0.76)	2.10 (1.74,2.54)	2.15 (1.78,2.61)
**Pre-pregnancy BMI**								
Underweight	0.95 (0.72,1.26)	0.89 (0.67,1.19)	1.21 (0.94,1.55)	1.28 (0.99,1.65)	1.68 (1.18,2.38)	1.69 (1.19,2.41)	0.64 (0.44,0.93)	0.67 (0.46,0.97)
Normal	1.00	1.00	1.00	1.00	1.00	1.00	1.00	1.00
Overweight	1.40 (1.13,1.75)	1.40 (1.12,1.75)	0.82 (0.63,1.08)	0.79 (0.60,1.04)	0.82 (0.53,1.26)	0.72 (0.46,1.12)	2.15 (1.72,2.69)	2.16 (1.73,2.70)
Obese	2.19 (1.61,2.98)	2.08 (1.51,2.86)	0.77 (0.48,1.25)	0.71 (0.43,1.15)	1.31 (0.74,2.34)	1.08 (0.60,1.95)	3.28 (2.40,4.48)	3.33 (2.43,4.56)
**Gestational weight gain (**0.1 kg/week)	1.09 (1.03,1.15)	1.10 (1.04,1.16)	1.14 (1.09,1.20)	1.13 (1.07,1.20)	0.84 (0.76,0.92)	0.78 (0.71,0.86)	1.31 (1.24,1.38)	1.32 (1.25,1.40)

^a ^method of delivery was three categories: normal, caesarean and other delivery methods. Multinomial logistic regression was used, considering normal delivery as the reference category

^b ^Preterm birth: binary outcome as normal vs. premature. Logistic regression was used, considering premature birth as an outcome

^c ^High risk: binary outcome as normal vs. high risk. Logistic regression was used, considering high risk as an outcome

^§ ^Fully adjusted model adjusted for maternal age, education, racial origin, cigarette smoking and alcohol consumption, birth weight, gestation and high risk pregnancy

^¥ ^Fully adjusted model adjusted for maternal age, education, racial origin, cigarette smoking and alcohol consumption and high risk pregnancy

**Table 2:** Mean difference (95% confidence interval) of birth weight (gm) (N = 6528) and placenta weight (gm) (N = 6281) by IOM, pre-pregnancy BMI categories and gestational weight gain per 0.1 kg/week, adjusting for potential confounding factors

	Mean difference of birth weight		Mean difference of placenta weight	
	Age adjusted	**Fully adjusted**^**¥**^	Age adjusted	**Fully adjusted**^**¥**^
**IOM^§^**				
Inadequate	-190.63 (-221.05,-160.20)	-152.82 (-180.34,-125.30)	-37.16 (-45.23,-29.09)	-37.80 (-45.87,-29.72)
Adequate (ref.)	0	0	0	0
Excess	206.45 (178.82,234.08)	211.34 (186.29,236.38)	41.16 (33.83,48.49)	42.76 (35.39,50.14)
**Pre-pregnancy BMI ^§^**				
Underweight	-207.26 (-249.21,-165.31)	-171.27 (-209.15,-133.38)	-43.23 (-54.18.-32.28)	-43.06 (-54.05,-32.08)
Normal (ref.)	0	0	0	00
Overweight	123.17 (84.47,161.87)	125.98 (90.97,161.00)	35.54 (25.47,45.61)	36.56 (26.44,46.67)
Obese	148.11 (85.32,21.091)	178.00 (121.09,234.91)	58.49 (41.97,75.00)	59.80 (43.20,76.41)
**Gestational weight gain^§ ^(**0.1 kg/week)	81.52 (72.97,90.07)	88.67 (81.09,96.29)	16.77 (14.52,19.01)	17.32 (15.05,19.59)

^¥^Fully adjusted model adjusted for maternal age, education, racial origin, cigarette smoking and alcohol consumption, gestation and high risk pregnancy

**^§^**IOM, pre-pregnancy BMI and gestational weight gain are considered three different exposures and analyses are performed separately for each of them.

**Table 3:** Mean difference (95% Confidence Interval) of length of stay in hospital from delivery to discharge by maternal BMI categories

Model numbers	N	Maternal BMI categories			
		
		Underweight	Normal	Overweight	Obese
Model 1	6528	0.00(-0.13,0.13)	0	0.01(-0.11,0.12)	0.30(0.10,0.49)
Model 2	6528	0.02(-0.10,0.15)	0	0.02(-0.10,0.14)	0.31(0.13,0.51)
Model 3	6528	0.04(-0.09,0.17)	0	-0.03(-0.15,0.09),	0.23(0.04,0.42)
Model 4	6528	0.07(-0.10,0.24)	0	0.00(-0.12,0.11)	0.07(-0.10,0.24)

Model 1: Adjusted for maternal age

Model 2: Adjusted for confounders: - maternal age, education, racial origin, cigarette smoking and alcohol consumption

Model 3: model 2+ adjusted for the mediating effects of complications of pregnancy

Model 4: model 3+ adjusted for the mediating effects of birth weights and method of delivery

**Table 4:** Mean difference (95% Confidence Interval) of length of stay in hospital from delivery to discharge by IMO recommendations

Model numbers		IOM categories (%)		
	**N**	**Inadequate** **(n = 1648)**	**Adequate** **(n = 2549)**	**Excess** **(n = 2324)**

Model 1	6521	-0.07 (-0.17,0.03)	0.0	0.19(0.10,0.27)
Model 2	6521	-0.07(-0.16,0.03)	0.0	0.20(0.11,0.29)
Model 3	6521	-0.05(-0.14,0.05)	0.0	0.15(0.07,0.24)
Model 4	6521	-0.07(-0.15,0.02)	0.0	0.16(0.08,0.24)

Model 1: Adjusted for maternal age

Model 2: confounder adjusted model - maternal age, education, racial origin, cigarette smoking and alcohol consumption

Model 3: model 2+ additionally adjusted for the mediating effects of complications of pregnancy

Model 4: model 3+ adjusted for the mediating effects of birth weights and method of delivery

## Results

On average, each mother gained 14.8 kg (SD 5.2) during her pregnancy, with an average of 0.4 kg per week (range: 0.0 to 0.9; SD 0.1) weight gain. Of the 6632 participants 1666 (25%) gained inadequate, 2571(39%) adequate and 2349 (36%) excessive weight during pregnancy according to IOM categories. 655 (9.9%) participants were underweight, 4924 (74.3%) had healthy weight, 778 (11.7%) were overweight and 275 (4.2%) were obese according to their pre-pregnancy BMI. 5079 (77.7%) mothers had normal delivery, 771 (11.8%) had caesarean delivery and the rest 678 (10.4%) had other deliveries including low forceps, mid forceps, ventouse, assisted breech, trial forceps and combined methods. Only 269 (4%) births delivered as premature and 582 (8.9%) had pregnancy complication. Mean birth weight was 3384.2 (SD 516.1) gm and placenta weight was 602.2 (SD131.5) gm. On average women stayed 4.3 (SD 1.6) days in the hospital from delivery to discharge. For normal vaginal delivery the mean length of hospital stay was 4.00 (SD 1.33) days, for caesarean delivery 6.21 (SD 1.58) days and for other types of delivery it was 4.80 (SD 1.55) days.

The unadjusted association of maternal characteristics with IOM categories are presented in additional file 1 table S2. Mothers with lower educational attainment, those of Aboriginal-Islander origin, and those who never smoked and abstained from alcohol prior to pregnancy were more likely than other women to gain excessive weight during pregnancy. Mothers who gained excessive weight were more likely to have experienced pregnancy complications, have had their infant delivered by caesarean section and to have had higher birth weight infants.

Table 1 shows odds of being caesarean and other deliveries compared to normal deliveries, preterm births compared to normal birth and high risk compared to normal risk pregnancy by IOM, pre-pregnancy BMI categories and gestational weight gain per 0.1 kg/week. The results are presented for the 6528 (91%) mothers with complete data on all variables included in the fully adjusted model. Model 1 shows maternal age adjusted odds ratios (OR) and model 2 shows OR adjusted for all other covariates. In the age adjusted model, mothers who gained excess weight during pregnancy were 1.29 (95% CI: 1.09, 1.54) times as likely to go for caesarean delivery compared to mothers who maintained healthy weight gain during pregnancy. Similarly, overweight mothers were 1.40 (1.13, 1.75) times and obese mother were 2.19 (1.61, 2.98) times as likely to experience caesarean delivery compared to their counterpart. For 0.1 kg/week increase of GWG, each mother was at 9% higher risk to experience caesarean delivery. Mothers who gained inadequate weight or were underweight before pregnancy, were at greater risk of delivering preterm and were at less risk if they gained excess weight. In contrast, those mothers who gained inadequate weight were at a decreased risk of developing pregnancy complications and those who gained excess weight were at greater risk of experiencing pregnancy complications. Similarly, for 0.1 kg/week increase of GWG the risk of preterm births was low, however, the risk was greater for experiencing pregnancy complications. All the association remain consistent adjusting for potential confounding factors.

Those mothers who did not gain adequate weight during pregnancy delivered a 190.63 (-221.05,-160.20) gm lighter baby and those who gained excess weight delivered a 206.45 (178.82, 234.08) gm heavier baby compared to mother who gained healthy weight (table 2). Similarly, mothers who were underweight before pregnancy delivered a lighter baby and overweight mothers delivered a heavier baby. For 0.1 kg/week increase of GWG, each mother delivered a 81.52 gm heavier baby. Similarly, for placenta weight those mothers who were underweight or did not gain adequate weight had lower placental weight, and conversely, it was greater if they gained excess weight or were overweight or obese.

Additional file 1, table S3 shows unadjusted mean length of hospital stay postnatally by pre-pregnancy BMI categories and IOM categories of weight gain. Excess pre-pregnancy BMI was associated with excess length of postnatal stay in hospital. These unadjusted results also show that excess weight gain in pregnancy is associated with excess length of postnatal stay.

The multivariable analyses for the association of pre-pregnancy BMI categories with length of postnatal hospital stay is presented in Table 3. In the age adjusted model (i.e. model 1), on average an obese mothers stayed 0.30 (0.10, 0.49) days longer in hospital postnatally compared to mothers with a healthy BMI. However, this association was attenuated with adjustment for potential confounders or mediators, particularly by adjustment for pregnancy complications and caesarean delivery.

Table 4 shows the multivariable analyses for the association of IOM categories of weight gain with length of postnatal hospital stay. Women who gained excessive weight during pregnancy (0.19 kg per day on average) stayed on average longer in hospital compared with those who gained adequate weight. These differences remained robust after adjustment for confounding factors (model 2) but were attenuated (approximately 20%) by adjustment for potential mediators (model 3). Since IOM categories combine pre-pregnancy BMI with weight gain categories no additional adjustment for pre-pregnancy BMI was made in these analyses.

Additional file 1, figure S1 shows mean differences in length of hospital stay from delivery to discharge by maternal weight gain per week per 0.1 kg/week during pregnancy. In the age adjusted model, a 0.1 kg/week excess maternal gestational weight gain was associated with 0.09 of a day (95% CI: 0.06, 0.11) longer hospital postnatal stay on average. There was **a **marginal attenuation after adjustments were made for the mediating effect of pregnancy complications (model 4).

In additional analyses, when we further adjusted the association of IOM or GWG with hospital stays by parity, maternal depression, family income and placenta weight, the associations remained unchanged (results available from author on request).

## Discussions

We found that mothers who gained excess weight during pregnancy were at greater risk of pregnancy complications, caesarean delivery, and had excess length of hospital stay. These women had larger babies and placentas. We also found that mothers who gained inadequate weight or were underweight before pregnancy were at greater risk of preterm birth, had lower rates of pregnancy complications, and had smaller infants and placentas. This association remained after adjustment for a range of potential confounding and mediating factors. In addition, consistent with other studies [14-17], we have found an association between pre-pregnancy obesity and increased length of hospital stay. However, in our study, adjustment for mediating factors attenuated this association towards the null.

Findings of pre-pregnancy obesity and excess weight gain associated with caesarean delivery [10], pregnancy complications [7,8,32], birth [12,13] and placenta weight [33] are consistent with previous studies. Our finding of no association between pre-pregnancy obesity and premature birth contrasted some studies [11] but remained consistent with others [34]. Findings of pre-pregnancy obesity associated with increased use of hospital stay are consistent with other studies [14-17]. Studies by Callaway et al [14] and Chu et al [15] found the association remained predominately for the morbidly obese (BMI > 40 kg/m^2^) group, but across the remainder of the BMI distribution the association of pre-pregnancy BMI with length of hospital stay was largely mediated by pregnancy complications. This difference could be due to the variations in different sample populations and measurements between our study and those of Callaway et al [14] and Chu et al [35].

To our knowledge no previous study has examined the association between GWG and length of postnatal hospital stay. We found for each 100 gm increase of GWG maternal stay in the hospital increased 0.09 days (i.e. mothers will stay 2.2 hours longer in the hospital, which is equivalent to one day longer stay in hospital for every one kg increase of GWG). The association of excess GWG and excess hospital stay found in our study could involve a pathway from excess weight gain during pregnancy to complications during pregnancy and caesarean section which in turn translate into a longer hospital stay. Alternatively, placenta praevia, placenta accreta and previous caesarean delivery might result in increased length of stay. Whilst there was some attenuation towards the null of the positive association between GWG and length of hospital stay in our study some independent association remained.

Beyond HDP and gestational diabetes we do not have information on other complications of pregnancy, such as placental abruption. However, HDP and gestational diabetes are the two key common pregnancy complications that will both affect weight gain in pregnancy and might result in increased length of hospital stay either because of the requirement for an operative delivery or because of persistent ill-health of the mother. HDP would potentially be associated with excess GWG because of maternal oedema with this condition and gestational diabetes would be potentially associated with excess GWG because of greater fetal growth associated with this condition. Despite adjusting for the two most common complications of pregnancy that are likely to mediate our association, it is possible that misclassification bias for both of these conditions means that we have not been able to fully adjust for their potential mediating effect. Since routine universal fasting blood glucose or oral glucose tolerance test were not used in this obstetric population to diagnose gestational diabetes it is possible that misclassification of some women with this condition as not having it means that we are not able to fully assess its role as a potential mediator. As we relied on routine clinical diagnoses of HDP it is also possible that misclassification of this condition limited our ability to fully adjust for its mediating effect. However, rather than limiting our classification to only include those with pre-eclampsia, our use of the broader category of HDP will reduce the likelihood that cases are missed.

In addition to the possibility that pregnancy complications explain a positive association between GWG and postnatal length of hospital stay, it is also possible that women who gain excess weight during pregnancy experience more postnatal complications, even without caesarean delivery, than women who gain adequate weight during pregnancy. This could include perineal tears, pressure sores, venous thrombosis and difficulties with breast feeding, each of which might lead them to a longer hospital stay. As we do not have information on postnatal events in this study, we were unable to test this possibility. However, a study by Sebire et al [9] showed that prep-pregnancy obesity was associated with all of these postnatal complications. Finally, the propensity to gain excessive weight during pregnancy might reflect underlying metabolic disturbances such as insulin resistance, which could result in postnatal complications and the need for a longer hospital stay. Again we were unable to explore this in our study.

Several limitations should be considered when interpreting the results. Our data represents the obstetric population of Brisbane in the early 1980's, this may not be representative of today's obstetric population. In contemporary obstetric populations, industrialised countries including Australia, United Kingdom and the United States of America, recommend short hospital stays (e.g. < 48 hours after birth) for healthy term newborns [36], which are considerably shorter than the average stay of 4 days in our study population. As expected the prevalence of overweight and obesity for the current obstetric population has increased two to three folds during the last three decades. During the same period caesarean section delivery has increased in the same hospital two to three folds (it was 11.8% in MUSP and now 27% [14]). Despite marked differences in mean length of postnatal hospital stay, prevalence of overweight/obesity and caesarean section delivery in our population compared to contemporary populations, the association of maternal pre-pregnancy BMI with length of stay in hospital and caesarean delivery are similar in MUSP to that of other contemporary populations [14,15] suggesting that the association may still be relevant. For instance, when we compared the association of pre-pregnancy obesity with length of hospital stay and caesarean delivery with the recent study by Callaway et al [14] who used obstetric data from11,252 women for the period 1998-2002 from the same hospital in Brisbane, the direction and magnitude of the associations reported are essentially the same in both studies. This suggests that our findings are likely to have relevance for today's obstetric population. In this study, weight gain in pregnancy is relatively crudely assessed since it relies on just two measurements and therefore we are unable to look at different patterns of weight change in pregnancy on length of hospital stay. However, our estimated mean rate of total gestational weight gain 0.38 (SD 0.14) is similar to the recent cohort study reported 0.39 kg/week (SD, 0.14) in the Project Viva [37].

## Conclusions

We found that pre-pregnancy obesity and excess weight gain during pregnancy were associated with greater odds of caesarean delivery and pregnancy complication, heavier birth and placenta weights. Excess GWG was associated with greater length of hospital stay independent of pre-pregnancy BMI, maternal life style, pregnancy complications and caesarean delivery. Inadequate GWG or pre-pregnancy underweight was associated with greater risk of preterm births. The relationship between pre-pregnancy obesity and increased length of hospital stay was fully mediated by pregnancy complications and caesarean delivery in this study population. Our results highlight the importance of routinely collecting accurate data on weight, height and weight gain throughout pregnancy, both to identify women at increased risk of health care requirements and so that other studies can replicate the results. In recent years, most high-income countries have seen a trend towards rapid discharge of mothers and babies after delivery in order to reduce the risk of hospital infection, improve rapid integration of the new-born into family life and provide a more efficient healthcare service. The main implication from this study is that, as well as causing adverse perinatal and longer term outcomes, excessive weight gain during pregnancy may also lead to adverse pregnancy outcomes and extended health care utilization in obstetric care. If our results are replicated in other cohorts, further research needs to determine the mechanisms linking these pathways of excess GWG to adverse pregnancy outcomes to longer hospital stay and identify means of supporting healthy weight gain in pregnancy.

## Disclosure of interests

The authors declare that they have no competing interests.

## Authors' contributions

AAM initiated the concept, conducted analyses and drafted the article. LKC and DAL critically revised the background and discussion of the manuscript. GMW assisted the statistical analyses and revised the methods of the article. MJO, JMN, RA and AC critically revised the whole manuscript. All authors read and approved the final manuscript.

## Pre-publication history

The pre-publication history for this paper can be accessed here:

http://www.biomedcentral.com/1471-2393/11/62/prepub

## Supplementary Material

Additional file 1 **Additional analyses conducted as part of the manuscript**. Three additional tables (tables S1, S2, and S3) and one additional figure (figure S1) as cited in the manuscript. Click here for file

## References

[B1] Obesity: preventing and managing the global epidemic Report of a WHO consultation World Health Organ Tech Rep Ser 2000 894 1 253 i-xii 11234459

[B2] Linne Y Effects of obesity on women's reproduction and complications during pregnancy Obes Rev 2004 5 3 137 43 10.1111/j.1467-789X.2004.00147.x 15245382

[B3] Cameron AJ Welborn TA Zimmet PZ Dunstan DW Owen N Salmon J Overweight and obesity in Australia: the 1999-2000 Australian Diabetes, Obesity and Lifestyle Study (AusDiab) Med J Aust 2003 178 9 427 32 12720507 10.5694/j.1326-5377.2004.tb05998.x

[B4] Troiano RP Flegal KM Kuczmarski RJ Campbell SM Johnson CL Overweight prevalence and trends for children and adolescents. The National Health and Nutrition Examination Surveys, 1963 to 1991 Arch Pediatr Adolesc Med 1995 149 10 1085 91 7550810 10.1001/archpedi.1995.02170230039005

[B5] Kumari AS Pregnancy outcome in women with morbid obesity Int J Gynaecol Obstet 2001 73 2 101 7 10.1016/S0020-7292(00)00391-X 11336728

[B6] Heslehurst N Ells LJ Simpson H Batterham A Wilkinson J Summerbell CD Trends in maternal obesity incidence rates, demographic predictors, and health inequalities in 36,821 women over a 15-year period Bjog 2007 114 2 187 94 17305899 10.1111/j.1471-0528.2006.01180.x

[B7] Garbaciak JA Jr Richter M Miller S Barton JJ Maternal weight and pregnancy complications Am J Obstet Gynecol 1985 152 2 238 45 4003470 10.1016/s0002-9378(85)80029-6

[B8] Guelinckx I Devlieger R Beckers K Vansant G Maternal obesity: pregnancy complications, gestational weight gain and nutrition Obes Rev 2008 9 2 140 50 10.1111/j.1467-789X.2007.00464.x 18221480

[B9] Sebire NJ Jolly M Harris JP Wadsworth J Joffe M Beard RW Maternal obesity and pregnancy outcome: a study of 287,213 pregnancies in London Int J Obes Relat Metab Disord 2001 25 8 1175 82 10.1038/sj.ijo.0801670 11477502

[B10] Durnwald CP Ehrenberg HM Mercer BM The impact of maternal obesity and weight gain on vaginal birth after cesarean section success Am J Obstet Gynecol 2004 191 3 954 7 10.1016/j.ajog.2004.05.051 15467571

[B11] McDonald SD Han Z Mulla S Beyene J Overweight and obesity in mothers and risk of preterm birth and low birth weight infants: systematic review and meta-analyses Bmj 341 c3428 10.1136/bmj.c3428 PMC2907482 20647282

[B12] Frederick IO Williams MA Sales AE Martin DP Killien M Pre-pregnancy body mass index, gestational weight gain, and other maternal characteristics in relation to infant birth weight Matern Child Health J 2008 12 5 557 67 10.1007/s10995-007-0276-2 17713848

[B13] Baker JL Michaelsen KF Rasmussen KM Sorensen TI Maternal prepregnant body mass index, duration of breastfeeding, and timing of complementary food introduction are associated with infant weight gain Am J Clin Nutr 2004 80 6 1579 88 15585772 10.1093/ajcn/80.6.1579

[B14] Callaway LK Prins JB Chang AM McIntyre HD The prevalence and impact of overweight and obesity in an Australian obstetric population Med J Aust 2006 184 2 56 9 16411868 10.5694/j.1326-5377.2006.tb00115.x

[B15] Chu SY Bachman DJ Callaghan WM Whitlock EP Dietz PM Berg CJ Association between obesity during pregnancy and increased use of health care N Engl J Med 2008 358 14 1444 53 10.1056/NEJMoa0706786 18385496

[B16] Galtier-Dereure F Boegner C Bringer J Obesity and pregnancy: complications and cost Am J Clin Nutr 2000 71 5 Suppl 1242S 8S 10799397 10.1093/ajcn/71.5.1242s

[B17] Galtier-Dereure F Montpeyroux F Boulot P Bringer J Jaffiol C Weight excess before pregnancy: complications and cost Int J Obes Relat Metab Disord 1995 19 7 443 8 8520632

[B18] National Health and Medical Research Council Clinical Practice Guidelines- for the management of overweight and obesity in children and adolescents 2003 Canberra: Commonwealth of Australia

[B19] Langnase K Mast M Danielzik S Spethmann C Muller MJ Socioeconomic gradients in body weight of German children reverse direction between the ages of 2 and 6 years 2003 133 3 789 96 10.1093/jn/133.3.789 12612154

[B20] Chu SY Callaghan WM Bish CL D'Angelo D Gestational weight gain by body mass index among US women delivering live births, 2004-2005: fueling future obesity Am J Obstet Gynecol 2009 200 3 271 e1 7 10.1016/j.ajog.2008.09.879 19136091

[B21] Hayatbakhsh MR Sadasivam S Mamun AA Najman JM Williams GM O'Callaghan MJ Maternal smoking during and after pregnancy and lung function in early adulthood: a prospective study Thorax 2009 64 9 810 4 10.1136/thx.2009.116301 19525264

[B22] Mamun AA O'Callaghan M Callaway L Williams G Najman J Lawlor DA Associations of gestational weight gain with offspring body mass index and blood pressure at 21 years of age: evidence from a birth cohort study Circulation 2009 119 13 1720 7 10.1161/CIRCULATIONAHA.108.813436 19307476

[B23] Nohr EA Vaeth M Baker JL Sorensen T Olsen J Rasmussen KM Combined associations of prepregnancy body mass index and gestational weight gain with the outcome of pregnancy Am J Clin Nutr 2008 87 6 1750 9 18541565 10.1093/ajcn/87.6.1750

[B24] Keeping JD Najman JM Morrison J Western JS Andersen MJ Williams GM A prospective longitudinal study of social, psychological and obstetric factors in pregnancy: response rates and demographic characteristics of the 8556 respondents Br J Obstet Gynaecol 1989 96 3 289 97 10.1111/j.1471-0528.1989.tb02388.x 2713287

[B25] Najman JM Bor W O'Callaghan M Williams GM Aird R Shuttlewood G Cohort Profile: The Mater-University of Queensland Study of Pregnancy (MUSP) International Journal of Epidemiology 2005 34 5 992 997 10.1093/ije/dyi119 15951358

[B26] World Health Organization Obesity. Preventing and Managing the Global Epidemic. Report of a WHO Consultation on Obesity, 3-5 June 1997 1998 Geneva, Switzerland.: World health Organization 11234459

[B27] Burke V Simmer K Oddy WH Blake KV Doherty D Kendall GE Newnham JP Landau LI Stanley FJ Predictors of body mass index and associations with cardiovascular risk factors in Australian children: a prospective cohort study International Journal of Obesity 2004 1 9 10.1038/sj.ijo.0802750 15314630

[B28] IOM (Institute of Medicine) Weight Gain During Pregnancy: Reexamining the Guidelines Washington, DC: The National Academies Press Posted online May 28, 2009 20669500

[B29] Callaway LK Lawlor DA O'Callaghan M Williams GM Najman JM McIntyre HD Diabetes mellitus in the 21 years after a pregnancy that was complicated by hypertension: findings from a prospective cohort study Am J Obstet Gynecol 2007 197 5 492 e1 7 10.1016/j.ajog.2007.03.033 17980185

[B30] Hernan MA Hernandez-Diaz S Werler MM Mitchell AA Causal knowledge as a prerequisite for confounding evaluation: an application to birth defects epidemiology Am J Epidemiol 2002 155 2 176 84 10.1093/aje/155.2.176 11790682

[B31] Bedford A Foulds GA Delusions Symptoms States Inventory: State of Anxiety and Depression (Manual) 1978 Berkshire, England: NFER Publishing

[B32] Fortner RT Pekow P Solomon CG Markenson G Chasan-Taber L Prepregnancy body mass index, gestational weight gain, and risk of hypertensive pregnancy among Latina women Am J Obstet Gynecol 2009 200 2 167 e1 7 10.1016/j.ajog.2008.08.021 19070831

[B33] Zhou W Olsen J Gestational weight gain as a predictor of birth and placenta weight according to pre-pregnancy body mass index Acta Obstet Gynecol Scand 1997 76 4 300 7 10.1111/j.1600-0412.1997.tb07982.x 9174421

[B34] Hauger MS Gibbons L Vik T Belizan JM Prepregnancy weight status and the risk of adverse pregnancy outcome Acta Obstet Gynecol Scand 2008 87 9 953 9 10.1080/00016340802303349 18720038

[B35] Schuurman N Peters PA Oliver LN Are obesity and physical activity clustered? A spatial analysis linked to residential density Obesity (Silver Spring) 2009 17 12 2202 9 10.1038/oby.2009.119 19390521

[B36] Hospital stay for healthy term newborns Pediatrics 2004 113 5 1434 6 15121968 10.1542/peds.113.5.1434

[B37] Oken E Kleinman KP Belfort MB Hammitt JK Gillman MW Associations of gestational weight gain with short- and longer-term maternal and child health outcomes Am J Epidemiol 2009 170 2 173 80 10.1093/aje/kwp101 19439579 PMC2727269

[B38] Magarey AMDL Boulton TJ Prevalence of overweight and obesity in Australian children and adolescents: reassessment of 1985 and 1995 data against new standard international definitions Medical Journal of Australia 2001 4 11 561 4 10.5694/j.1326-5377.2001.tb143435.x 11453327

