# Supplementary material for: Associations of maternal pre-pregnancy obesity and excess pregnancy weight gains with adverse pregnancy outcomes and length of hospital stay
Source: BMC Pregnancy Childbirth. 2011 Sep 6;11:62. doi: 10.1186/1471-2393-11-62 (PMC3178538; doi:10.1186/1471-2393-11-62)
Supplement: Additional file 1 — Additional analyses conducted as part of the manuscript. Three additional tables (tables S1, S2, and S3) and one additional figure (figure S1) as cited in the manuscript. [file 1471-2393-11-62-S1.DOC]

**ADDITIONAL FILE**

Title: Additional analyses conducted as part of the manuscript

Description: Three additional tables (additional tables 1, 2, and 3) and one additional figure as cited in the manuscript

**Table S1: Institute of Medicine categories** ^38^

Prepregnancy BMI (kg/m^2^) Gestational weight gain (kg)

- Adequate: <19.8 12.5-18.0

19.8-26.0 11.5-16.0 26.0-29.0 7-11.5

>29.0 6.0-11.5

- Inadequate: <19.8 <12.50

19.8-26.0 <11.5

26.0-29.0 <7.0

>29.0 <6.0

- Excess: <19.8 >18.0

19.8-26.0 >16.0

26.0-29.0 >11.5

>29.0 >11.5

**Table S2: Distributions of maternal characteristics by IOM categories of weight gain in pregnancy.**

| **Maternal factors** | **N** | **IOM categories (%)** | | |
| --- | --- | --- | --- | --- |
|  |  | Inadequate  (n=1666) | Adequate  (n=2571) | Excess  (n=2349) |
| **Education** |  |  |  |  |
| Did not complete secondary school | 1162 | 26.5 | 34.6 | 38.9 |
| Completed secondary school | 4253 | 24.6 | 40.0 | 35.5 |
| Higher education | 1171 | 26.8 | 40.1 | 33.1 |
| p-value |  | <0.001 | | |
| **Racial origin of parents** |  |  |  |  |
| White | 5787 | 24.7 | 39.7 | 35.6 |
| Asian | 270 | 36.7 | 41.9 | 21.5 |
| Aboriginal-Islanders | 384 | 26.8 | 31.3 | 41.9 |
| **p-value** |  | <0.001 | | |
| **Maternal smoking before pregnancy** |  |  |  |  |
| Never | 3324 | 26.1 | 39.9 | 34.0 |
| 1-19 cigarettes per day | 1971 | 23.5 | 39.5 | 37.2 |
| 20 or more cigarettes per day | 1287 | 26.3 | 36.0 | 37.7 |
| P-value |  | 0.009 | | |
| **Maternal alcohol before pregnancy** |  |  |  |  |
| Abstainer | 1636 | 26.5 | 36.7 | 36.7 |
| Light | 4312 | 25.1 | 40.1 | 34.8 |
| 1+per day | 638 | 23.2 | 37.9 | 38.9 |
| p-value |  | 0.056 | | |
| **Maternal depression** |  |  |  |  |
| Non-depressed | 6144 | 25.4 | 39.0 | 35.6 |
| Depressed | 362 | 25.1 | 39.0 | 35.9 |
| p-value |  | 0.992 | | |
| **Method of delivery** |  |  |  |  |
| Normal | 5150 | 26.5 | 39.5 | 34.0 |
| Lower segment caesarean delivery | 791 | 21.7 | 37.2 | 41.1 |
| Others | 687 | 20.4 | 38.1 | 41.5 |
| **Gestation** |  | <0.001 | | |
| Normal | 6363 | 24.4 | 39.3 | 36.3 |
| Premature | 269 | 46.8 | 33.1 | 20.1 |
| p-value |  | <0.001 | | |
| **Pregnancy complications** |  |  |  |  |
| No | 6029 | 26.6 | 39.8 | 33.6 |
| Yes | 595 | 12.3 | 32.3 | 55.6 |
| p-value |  | <0.001 | | |
| Mean (SD) maternal age in yrs | 6632 | 25.1(5.1) | 25.2(5.0) | 24.7(5.1) |
| p-value |  | <0.001 | | |
| Mean (SD) birthweight in grams | 6632 | 3170.6(503.7) | 3361.3(487.8) | 3560.8(493.2) |
| p-value |  | <0.001 | | |
| Mean (SD) placenta weight in grams | 6386 | 559.6(121.1) | 597.3(128.0) | 637.5(132.8) |
| p-value |  | <0.001 | | |

**Table S3: Mean post-natal hospital stay by categories of maternal pre-pregnancy BMI and IOM categories**

|  | N (%) | Mean number of days from delivery to discharge | 95% Confidence Interval |
| --- | --- | --- | --- |
| **Pre-pregnancy BMI categories** |  |  |  |
| Underweight | 655 (10) | 4.35 | 4.22, 4.47 |
| Normal | 4924 (74) | 4.33 | 4.29, 4.38 |
| Overweight | 778 (12) | 4.33 | 4.21, 4.44 |
| Obese | 275 (4) | 4.61 | 4.39, 4.83 |
| p-value |  | 0.039 |  |
| **Institute of Medicine category** |  |  |  |
| Adequate | 2591 (39) | 4.30 | 4.24, 4.35 |
| Inadequate | 1679 (25) | 4.22 | 4.15, 4.30 |
| Excess | 2362 (36) | 4.50 | 4.42, 4.55 |
| p-value |  | <0.001 |  |

F**igure S1: Mean difference (95% Confidence Interval) of length of stay in hospital from delivery to discharge by maternal weight gain during pregnancy (N=6528)**

Model 1: Adjusted for maternal age

Model 2: model 1 + additionally adjusted for maternal BMI

Model 3: model 2 + additionally adjusted for confounding factors maternal education, cigarette smoking and alcohol consumption

Model 4: model 3+ additionally adjusted for the mediating effects of complications of pregnancy

Model 5: model 4+ additionally adjusted for the mediating effects of birth weights and method of delivery
